# Alkane-induced expression, substrate binding profile, and immunolocalization of a cytochrome P450 encoded on the *nifD *excision element of *Anabaena *7120

**DOI:** 10.1186/1471-2180-5-16

**Published:** 2005-03-24

**Authors:** Sergio Torres, Conrad R Fjetland, Peter J Lammers

**Affiliations:** 1 Department of Chemistry and Biochemistry, New Mexico State University, Las Cruces, NM, USA; 2 University of Texas at Austin, Department of Chemistry and Biochemistry, Austin, TX, USA

## Abstract

**Background:**

Alkanes have been hypothesized to act as universal inducers of bacterial cytochrome P450 gene expression. We tested this hypothesis on an unusual P450 gene (*cyp110*) found on a conserved 11 kilobase episomal DNA element of unknown function found in filamentous cyanobacteria. We also monitored the binding of potential substrates to the P450 protein and explored the distribution of P450 protein in vegetative cells and nitrogen-fixing heterocysts using immuno-electron microscopy.

**Results:**

Hexadecane treatments resulted in a two-fold increase in mRNA, and a four-fold increase in P450 protein levels relative to control cultures. Hexane, octane and dodecane were toxic and induced substantial changes in membrane morphology. Long-chain saturated and unsaturated fatty acids were shown to bind the CYP110 protein using a spectroscopic spin-shift assay, but alkanes did not bind. CYP110 protein was detected in vegetative cells but not in differentiated heterocysts where nitrogen fixation occurs.

**Conclusion:**

Hexadecane treatment was an effective inducer of CYP110 expression in cyanobacteria. Based on substrate binding profiles and amino acid sequence similarities it is hypothesized that CYP110 is a fatty acid ω-hydroxylase in photosynthetic cells. CYP110 was found associated with membrane fractions unlike other soluble microbial P450 proteins, and in this regard CYP110 more closely resembles eukarytotic P450s. Substrate stablization is an unlikely mechanism for alkane induction because alkanes did not bind to purified CYP110 protein.

## Background

*Anabaena *sp. strain PCC 7120 (*Anabaena *7120) is an obligate photoautotrophic cyanobacterium that reduces atmospheric nitrogen in terminally differentiated cells known as heterocysts [1]. Approximately every tenth cell along a filament differentiates into a heterocyst under nitrogen-fixing conditions. Three developmentally regulated DNA rearrangements occur within the heterocyst chromosome which excise DNA elements integrated within the *nifD*, *fdxN *and *hupL *genes [2,3]. The element interrupting the *nifD *gene is 11,268 base pairs in length and carries its own site-specific recombinase, the *xisA *gene [4]. The *nifD *element is found in most heterocyst forming cyanobacteria integrated within the *nifD *gene [5,6]. It is absent from all non-heterocystous strains examined to date [7,8]. While factors driving the evolution of the *nifD *element and its associated genes remain obscure, an essential role in diazotrophic growth can be ruled out since an *Anabaena *strain cured of the *nifD *element was shown to fix nitrogen under aerobic conditions in heterocysts of normal morphology [9].

**Figure 1:**
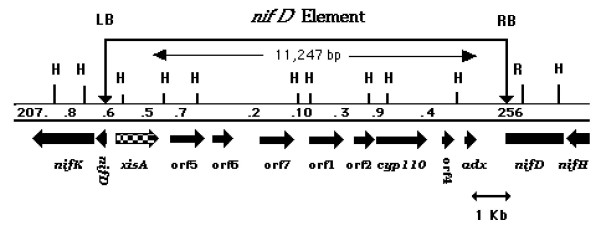
**Genetic Map of the *nif D *Element**. The positions of known *nif D *element genes and other open reading frames are indicated with arrows showing the orientation. LB = left border, RB = right border, H = *Hin*dIII sites. The numbers inside the double lines designate the *Hin*dIII fragment subclones of the *nifD *element derived from a 17 kbp *Eco*RI fragment, An 207. The *cyp110 *probe was made from the An207.4 *Hin*dIII fragment. The complete sequence of the *nif D *element is deposited in Genbank (U38537).

The complete DNA sequence of the *nifD *element reveals little about the biological functions it encodes. Nine open reading frames are known (see Fig. 1) only one of which can be identified by sequence homology: the first reported cyanobacterial cytochrome P450 gene, *cyp110 *[10]. The *cyp110 *gene is widely conserved in the heterocyst forming cyanobacteria including *Anabaena*, *Nostoc *and *Calothrix/Fremyella*. The amino acid sequence of CYP110 is most similar to the mammalian P450 family 4 fatty acid omega-hydroxylases and P450_BM3 _from *Bacillus megatarium*, another fatty acid omega hydroxylase. However, the metabolic significance of fatty acid omega hydroxylation in bacteria remains obscure. Even for the well-characterized P450_BM3 _enzyme, the identity of the *in vivo *substrate(s) of this protein and the pathway involved are not known [11]. Studies suggest n-alkanes act as multi-purpose microbial P450 inducers and our preliminary data suggested this was also true for *cyp110 *[12,13]. Moreover, a biosensor for hexadecane detection based on changes in *cyp110 *mRNA from *Anabeana variabilias *has been described [14]. Here we report on the pattern of expression of the *cyp110 *mRNA and protein in *Anabaena *7120, substrate binding characteristics of CYP110, the effects of alkanes on cellular morphology, and the distribution of CYP110 in vegetative cells versus heterocysts using electron microscopy.

## Results

### Hexadecane-dependent induction of *cyp110 *transcripts in nitrogen-fixing and ammonia-supplemented cultures

We first tested the effects of hexane, octane, dodecane and hexadecane on the growth of *Anabaena *7120 cultures. All alkane additions in this study were carried out at 0.2% (v/v), the recommended concentration for microbial P450 induction [12]. In preliminary experiments, we determined that *Anabaena *7120 exhibited rapid chlorosis with 0.2% hexane and octane. These treatments yielded only highly degraded RNA and were not further analyzed (data not shown). RNA samples from control, dodecane and hexadecane treated cultures were size fractionated and hybridized with *cyp110*. A *psbA1 *specific probe was used to assess RNA quality and for normalization.

**Figure 2:**
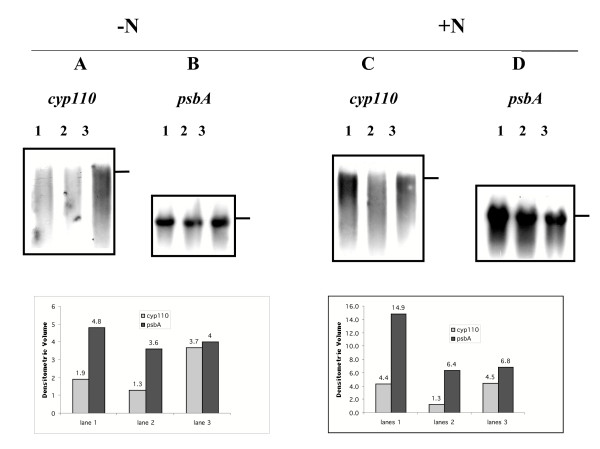
**Expression of *cyp110 *and *psbA *transcripts in nitrogen-fixing and ammonia-supplemented *Anabaena *7120 cultures**. *Anabaena *7120 control and test cultures were grown in nitrogen-free (-N) and ammonia-supplemented (+N) Chu #10 media. Test cultures were treated with 0.2 % (v/v) dodecane or hexadecane for 2 hours. Fifteen microgram samples of total *Anabaena *7120 RNA from each treatment were size fractionated, transferred to a nylon membrane and probed with a *cyp110 *probe (panels A and C). RNA blots were stripped and reprobed with a *psbA *probe (panels B and D). Lanes (1) untreated control, lanes (2) dodecane treatment, lanes (3) hexadecane treatment. Bars on left side of each panel denote the approximate transcript sizes (A, and C = 8.5 kb, B and D = 1.3 kb). Quantification data is shown below each northern blot.

The *cyp110 *probe identified a range of transcript sizes with a maximum of 9 kb seen in mRNA from both nitrogen-fixing and ammonia supplemented *Anabaena *7120 cultures Fig. 2(a) and 2(c). The size of the transcript identified by the *cyp110 *probe and the genetic organization of the *nifD*-element suggest that all of the open reading frames to the right of *xisA *on the *nifD *element are coordinately transcribed on a polycistronic mRNA. In support of this idea, a probe internal to the ORF6 coding region (Fig. 1) identified the same range of transcript sizes (data not shown). Since the *xisA *mRNA has not been detected with Northern blots [9], it is likely that the 9 kb transcript begins downstream of the *xisA *gene and terminates 3' to the orf designated *adx *in Fig. 1.

The *psbA1 *gene, which encodes the D1 reaction center protein, was used as a load control and hybridization standard. As seen in Fig. 2(b) and 2(d), the *psbA1 *probe identifies a discrete 1.25 kb transcript in control RNA as previously shown [15]. Relative amounts of mRNAs were estimated by densitometry of the scanned autoradiogram. Exposure times for detection of the *cyp110 *transcript were 12 times longer than for the highly expressed *psbA1 *mRNA.

The ratio of the *cyp110/psbA1 *signal intensities was 2.3 fold higher in hexadecane-treated cultures in the nitrogen-fixing culture (panels A&B) while a 2.2-fold increase was observed in the ammonium-supplemented culture (panels C&D). The dodecane treatment had little effect on *cyp110 *mRNA levels in either nitrogen-fixing or ammonium supplemented cultures. Similar results were obtained in 2 separate experiments with RNA isolated from independent cultures. The doubling of steady-state *cyp110 *mRNA levels in response to hexadecane treatment was independent of nitrogen status of the culture, consistent with previous data showing the entire *nifD *element is not required for nitrogen fixation in heterocysts [9].

### CYP110 protein is only detected in hexadecane-treated cultures and localized in the membrane fraction

**Figure 3:**
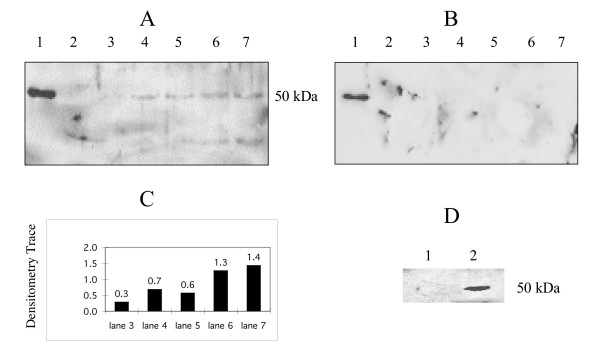
**Expression of CYP110 protein in *Anabaena *7120**. Proteins were fractionated on 12% polyacrylamide gels, blotted to nitrocellulose and probed with rabbit anti-CYP110 antibody. Panel A displays samples treated with 0.2 % n-hexadecane. Panel B, control untreated samples taken at the same time points. Lanes 1 contain 600 ng of purified recombinant CYP110 protein. Lanes 2 are blank. Lanes 3 through 7 contain 50 μg of crude protein taken at 0, 2, 12, 24, and 36 hours. Panel C shows the densitometry trace volumes for the CYP110 protein in Panel A. Panel D is a Western blot showing *cyp110*-reactive proteins from the 36 hour hexadecane time point after separation into the soluble and membrane fractions. Lane 1: 50 μg of the cytosolic fraction and lane 4: 50 μg of the membrane fraction.

The hexadecane effect on *cyp110 *gene expression was further examined using CYP110-specific antibodies to probe for the presence of P450 protein in nitrogen-fixing *Anabaena *7120 cultures. As shown in Fig. 3A, the 0.2% hexadecane treatment resulted in the appearance of an immuno-reactive protein band of 50 kDa that co-migrated with a purified CYP110 protein sample. The P450 protein band intensity increased at least 2 fold within 2 hours of treatment, and at least 4 fold 24 hours after treatment with hexadecane when compared to the background, zero-time expression shown in lane 3 (Fig. 3C). A western blot with protein samples from an untreated control culture failed to detect any immuno-reactive P450 protein (Fig. 3B). These results were replicated with 2 independent cultures.

Heterologous expression of the CYP110 protein in *E. coli *produced a substantial amount of membrane associated P450 protein [16]. This is a clear distinction between CYP110 and its closest sequence homolog, P450_BM3_, which has been extensively studied in part because it is a soluble P450 model protein [11]. To determine if CYP110 is membrane associated in *Anabaena *7120, protein from membrane and soluble fractions separated by centrifugation were subjected to Western blot analysis. As shown in Fig. 3D, the membrane fraction appeared to contain all of the immuno-reactive P450 protein in the *Anabaena *7120. This stands in contrast to P450_BM3 _and most other microbial P450 proteins, and in this regard, CYP110 more closely resembles the eukaryotic P450 proteins.

### Spectral characterization of CYP110 protein and substrate binding assay

**Figure 4:**
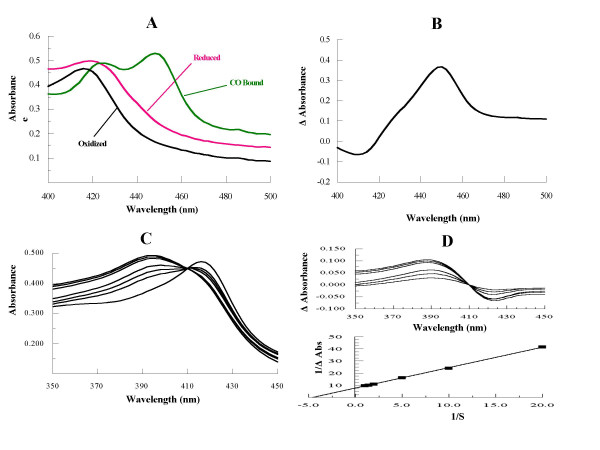
**CYP110 Absorbance Spectra, Titrated Spectral Shift, and Double Reciprocal Plot in the presence and absence of various hydrocarbons**. The absorbance characteristics were monitored for the purified soluble CYP110 from *E. coli*. The sample was diluted 1:5 in buffer A with 0.2 % deoxycholate. To reduce the P450, 5 mg of sodium dithionate was added to the sample. Carbon monoxide was then bubbled into the sample for 30 seconds. Panel A. Oxidized, reduced and carbon monoxide bound spectra. Panel B. Carbon monoxide difference spectrum with a peak at 450 nm. Panel C. The oxidized spectra for 3 μM CYP110 in buffer A + 0.2 % deoxycholate and titrated with tetradecanoic acid. The concentrations of tetradecanoic acid shown are 0, 0.1, 0.2, 0.5, 0.7, and 1.0 mM. Panel D. (Top) The difference spectra generated for CYP110 when titrated with tetradecanoic acid. The bottom plot for Panel D shows the spectrum without fatty acid subtracted from each spectra with fatty acid. The double reciprical plot of 1/ΔAbs vs 1/S is shown and the X-intercept is calculated by linear regression. Ks is defined as -1/X-intercept. ΔAbs for each fatty acid concentration is defined as the resulting value at 388 nm.

Absorption spectra studies were recorded for CYP110 protein expressed and purified from *E. coli *carrying a pUC-based expression vector [16]. As shown in Fig. 4A, the CYP110 protein has a typical cytochrome P450 absorbance profile in the visible region for the oxidized, reduced and carbon monoxide bound states. CYP110 also shows the characteristic peak at 450 nm in the CO difference spectrum (Fig. 4B). We monitored the visible spectrum while titrating a CYP110 solution with tetradecanoic acid. As shown in Fig. 4C, this resulted in a blue shift of the peak absorbance in the oxidized spectrum to 388 nm, similar to previously described P450 substrate-dependent Type I spectral shifts. This spectral shift was the same in either buffer A or buffer A + 0.2% deoxycholate. When buffer A was supplemented with 0.2% Triton X-100, no spectral shift was observed (data not shown). The titration yields the binding constant (Ks) for each substrate producing a spectral shift. A typical difference spectrum generated from the titration and the resulting double reciprocal plot are shown in Fig. 4D.

The calculated binding constants for the interaction of CYP110 with potential substrate molecules of different hydrocarbon chain length are listed in Table 1. For the saturated fatty acids tested, CYP110 displays the highest affinity for tetradecanoic acid with a K_s _of 0.091 mM. The unsaturated fatty acids also bound well to CYP110. Overall, CYP110 had the highest affinity for alpha-linolenic acid (Ks 0.062 mM) and arachidonic acid (Ks = 0.051 mM), long chain fatty acids with 3 and 4 double bonds, respectively. For comparison, P450_BM3 _has a K_s _of 0.002 mM for arachidonic acid [17]. The Ks values towards arachidonic acid for the mammalian cytochrome P450s from family 4 are in the same range [18].

Fatty amines and fatty amides are known to interact with P450 ω-hydroxylases via Type II spectral shifts [19,20], and this was also true for CYP110. The minimal chain length for this interaction was 5 since n-butylamine did not cause a spectral shift while n-pentylamine did bind. There was a clear trend for tighter binding with longer hydrocarbon chain length, as the CYP110-dodecylamine interaction had the lowest tested Ks value (0.012 mM). CYP110 bound fatty amines 10 fold better than P450_non_, which has a binding constant of 1.3 mM towards octylamine [21]. Twelve-aminododecanoic acid causes a Type I spectral shift instead of a Type II suggesting an altered mode of binding. Lu *et al*. [20] showed that terminal amino fatty acid derivatives interact weakly with the heme iron of cytochromes P450.

The binding of CYP110 towards other fatty acid derivatives was also examined. Table 1 displays a list of compounds that displayed no spectral shift, including fatty alcohols or saturated n-alkanes. No spectral shift was observed with 11- or 12-hydroxydodecanoic acid or with methyl laurate. The unsaturated fatty acid 11-dodecenoic acid was also tested with no shift observed. Two other amines that did not display spectral shifts were the polyamine, putrescine, and cetyltrimethylammonium bromide (CTAB). Sodium dodecyl sulfate (SDS) does bind CYP110 and causes a Type I spectral shift. Although no binding constant was determined, it was observed that the maximal shift to 388 nm occurred below 0.5 mM and reverted back to 416 nm above this concentration, a phenomenon likely to be linked to the critical micelle concentration for SDS (data not shown).

### Alkane effects on morphology and localization of CYP110

**Figure 5:**
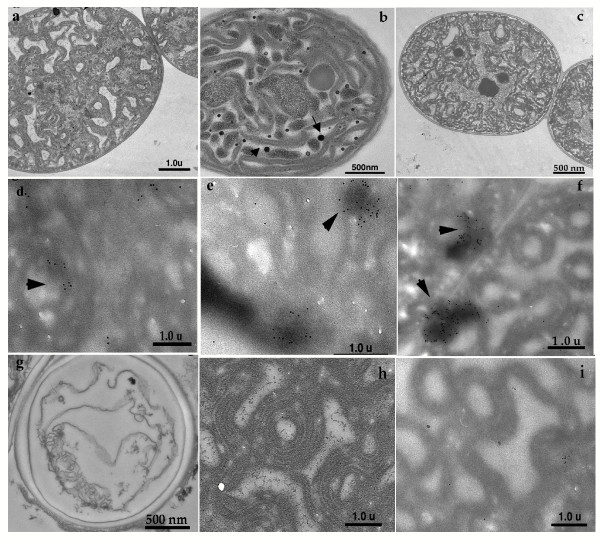
**Ultrastructure and immunocytochemical detection of CYP110 in alkane treated *Anabaena *7120**. Nitrogen-fixing cultures were grown with shaking in continuous light at 30 C with ambient CO_2_. Test cultures were treated with 0.2% dodecane or 0.2% hexadecane for 30 days. (a) TEM of untreated vegetative cell, (b) TEM of dodecane-treated vegetative cell, (c) TEM of hexadecane-treated vegetative cell, (d) untreated control vegetative cell incubated with anti-CYP110 antiserum, (e) dodecane-treated vegetative cell incubated with anti-Cyp111 antiserum, (f) hexadecane-treated vegetative cell incubated with anti-CYP110 antiserum, (g) hexadecane treated heterocyst incubated with anti-CYP110 antiserum, (h) dodecane-treated vegetative cell incubated with non-immune rabbit IgG, (i) dodecane-treated vegetative cell incubated with anti-CYP110 antiserum previously adsorbed with purified CYP110.

Given the toxicity of hexane to *Anabaena *7120, transmission electron microscopy was used to document changes in cell structure associated with the alkane treatments shown to induce the CYP110 expression. Vegetative cells from control, dodecane and hexadecane treated cultures are shown in Fig. 5a,5b and 5c respectively. Many dark spherical inclusions are seen in the dodecane-treated cells (Fig. 5b). The dodecane treatment also distorts and thickens the cellular and photosynthetic membranes, creating a ruffled effect in both outer and inner membranes. Hexadecane treatment did not have the same dramatic effect on membrane morphology, however the close stacking of photosynthetic membranes seen in the control micrograph (Fig. 5a) is lost with hexadecane treatment (Fig. 4c).

Gold-conjugated anti-CYP110 antibodies were used to determine the relative protein expression levels in vegetative cells with and without alkane treatment and to assess vegetative cell versus heterocysts expression patterns *Anabaena *7120. Immunoelectron micrographs showed a greater number of anti-CYP110 polyclonal gold conjugates in dodecane and hexadecane vegetative cells (Fig. 5e and 5f) than in control samples (Fig. 5d). Expression levels were quantified by counting gold particles in multiple micrographs as shown in Table 2. Immunogold particles most often clustered around dark-staining material in *Anabaena *7120 vegetative cells grown in the presence of 0.2% (v/v) dodecane or hexadecane (Fig. 5e,f). We found fewer immunogold particles/cluster in dodecane-treated samples (avg. = 22) than hexadecane treated samples (avg. = 50). Heterocysts showed significantly fewer gold particles than vegetative cells, and the number of gold particles in heterocysts did not increase with hexadecane treatment (Fig. 5g, Table 2).

Nonspecific immuno-staining was estimated by incubating sections with preimmune rabbit IgG or with polyclonal anti-CYP110 antibodies that had been pre-adsorbed with purified CYP110 protein produced in *E. coli*. Very low background was seen in both the preimmune rabbit IgG primary incubation (Fig. 5h), and sections treated with preabsorbed anti-CYP110 antibodies (Fig. 5i). No clusters of immunogold conjugates were seen in any of the control sections. These results clearly show the majority of CYP110 protein is localized in vegetative cells and corroborate Northern and Western data showing elevated CYP110 expression in hexadecane-treated cultures.

## Discussion

Most well characterized microbial P450s enzymes were isolated from heterotrophic organisms and are involved in the oxidative metabolism of xenobiotic compounds for energy and growth. Examples include camphor-degrading P450_CAM _[22] and two herbicide inducible P450 genes in *Streptomyces griseolus *[23]. There is no evidence to date for cytochrome P450-dependent oxidative metabolism in the cyanobacteria. A P450-dependent *N-*demethylation activity is known in unicellular green algae that activates the pro-herbicide metflurazon into norflurazon [24]. Two genera of marine cyanobacteria *Microcoleus *and *Phormidium *were isolated from oil spills along the Arabian sea and reported to contribute to oil degradation [25]. While the first step in alkane degradation in many microbial systems is a P450-dependent hydroxylation [11,26,27], there are no data on the mode of alkane metabolism nor confirmation of hydrocarbon oxidation by axenic isolates of these cyanobacterial strains. Recent evidence does show that mixtures of these cyanobacteria and organotrophic bacteria of the genera *Rhodococcus*, *Arthrobacter*, *Pseudomonas*, and *Bacillus *are more effective than either group alone towards alkane oxidation in inorganic growth media [28].

The available evidence suggests that CYP110 does not participate in the degradation of alkanes. CYP110 has low overall sequence similarity to other known alkane hydroxylase enzymes like the CYP52 family [29]. Alkanes are quite toxic to *Anabaena *7120, and purified CYP110 protein expressed in *E. coli *binds fatty acids but not alkanes using a spin-shift assay. The membrane localization and spin-shift binding data suggest that long-chain polyunsaturated fatty acids are the most likely endogenous substrates for CYP110. The endogenous function of CYP110 in *Anabaena *7120 remains a mystery. Sequence comparisons indicate the protein is related to the fatty acid omega hydroxylase proteins. One possibility is that ω-hydroxylated fatty acids are further oxidized to dicarboxylic acids which then undergo β-oxidation from both ends yielding acetate, as demonstrated in eukaryotic peroxisomes [30].

Cytochrome P450 proteins in diazotrophic bacteria have been suggested to play an ancillary role in nitrogen fixation. Many years ago, Appleby proposed that a cytochrome P450 enzyme functions as an alternative high-affinity oxidase in an efficient, leghemoglobin-facilitated pathway of oxidative phosphorylation in bacteroids of *Bradyrhizobium japonicum *[31]. The idea was based on spectroscopic evidence for the presence of a P450 protein in *B. japonicum *bacteroids but not in aerobically grown cells and the blocking of the high-affinity oxidase system by the P450 inhibitor, N-phenylimidazole. More recently both bacteroids and anaerobically grown *B. japonicum *cells were shown to contain two immunologically distinct cytochromes P450, which were cloned and designated *cyp112 *and *cyp114 *[32]. Neither protein is expressed in aerobically grown *B. japonicum *cells, consistent with the Appleby hypothesis. Transposon mutagenesis of the *cyp112 *locus was found to block expression of both Cyp112 and Cyp114. Nevertheless, the mutant strain produced effective nodules on soybeans. These results show neither protein is essential for symbiotic function under the conditions of plant growth used in these experiments. Subsequent analysis of the *B. japonicum *P450 cluster identified a third P450 gene and other open reading frames suggesting an operon involved in terpenoid biosynthesis [33]. Like the *Anabaena *7120 strain missing the *nifD *element, a quantitative phenotype related to nitrogen fixation under specific culture conditions cannot be excluded from the available evidence. *B. japonicum *and *Anabaena *7120 are not the only diazotrophic bacteria harboring cytochrome P450 genes near clusters of nitrogen fixation-related genes. The large Sym plasmid from *Rhizobium sp. NGR234 *confers the ability to associate symbiotically with plants. It contains two cytochrome P450 genes whose function(s) are not known [34].

## Conclusion

Alkanes have been hypothesized to act as universal inducers of microbial P450 genes [12]. Consistent with this hypothesis, we observed a small but reproducible 2-fold enhancement of *cyp110 *transcription induced by 0.2% hexadecane. We did not test lower concentrations of hexane or dodecane to determine if non-toxic concentrations would have P450-inducing activity in *Anabaena *7120. We cannot rule out the possibility that the lower solubility of longer chain alkanes in aqueous solutions may have been more important for the activity of hexadecane as an inducer than chain-length *per se*. The CYP110 protein displayed the highest affinity towards long-chain unsaturated fatty acids and was localized primarily in photosynthetic vegetative cells of *Anabaena *7120. Unlike most prokaryotic P450 proteins, CYP110 was found to be associated with membrane fractions consistent with the solubility characteristics of its preferred substrates.

## Methods

### Cyanobacterial growth

For RNA and protein isolation, cultures of *Anabaena *7120 (4 L) were grown in Chu #10 media with continuous light, stirred at 30°C and bubbled with a 1% CO_2_/air mixture illuminated at approximately 100 μE s^-1 ^m^-1^. Nitrogen supplemented cultures were grown with 2.5 mM NH_4_(SO_4_)_2 _to suppress heterocyst differentiation. Treatments were carried out after cultures reached A_700 _= 0.7. Control and test cultures (0.2 % dodecane or hexadecane [v/v] for 2 hours) were maintained in the same growth conditions during the treatment interval.

### RNA methods

An amended RNA isolation procedure [37] was used for total RNA isolation. Briefly, cells were cooled by adding an equal volume of ice followed by transferring the cell paste into a mortar and freezing with liquid nitrogen. Cells were ground to a fine powder and placed in 10 mL RNA lysis buffer-pH 5.2 (0.1 M Tris, 0.1 M LiCl, 5 mM EDTA, 0.1 M NaCl, 0.1 M sodium acetate, 1 % SDS, 0.2% β-mercaptoethanol). After vortexing (2 min), 5 mL hot (60 C) NTE saturated-phenol, pH 6.0, was added, followed by 3 min of vortexing. Samples were placed at 60°C for 10 min, vortexed and 7 mL chloroform:isoamyl alcohol (24:1) added and the aqueous phase was subjected to repeated phenol/chloroform extractions until no visible material remained at the interface. Total RNA was precipitated overnight in 2 M LiCl at 4°C. RNA pellets were washed with 2 M LiCl and ethanol precipitated. After a 70% ethanol wash, the RNA was brought up in diethyl pyrocarbonate treated water.

Samples of total RNA (15 μg) were separated by agarose formaldehyde denaturing gel electrophoresis. The gel was equilibrated twice in 10× SSC for 15 min and RNA was transferred onto a nylon membrane (Boehringer Mannheim) by capillary transfer. Probes were labeled using the DIG-High Prime Mix (Boehringer Mannheim) following the manufactures' recommendations. The *cyp110 *probe was derived from the 2 kbp *HindIII *pAn 207.4 DNA fragment (Fig. 1) containing most of the *cyp110 *gene sequence [10]. The *psbA *probe used is a 2 kbp pAn 625 *Eco*R1 fragment [15] containing the coding region for the D1 reaction center protein in photosystem II. RNA blots were first probed with *cyp110*, stripped, and reprobed with the *psbA *probe. The hybridized probes were detected using the CDP-*Star *(Boehringer Mannheim) chemiluminescent substrate, followed by autoradiography. Prehybridizations, hybridizations, and washes were carried out as described by the manufacturer. Signal intensities from northern blots were quantified using Intelligent Quantification software (Genomic Solutions).

### P450 protein analysis

Proteins for Western blotting were isolated from N_2_-fixing *Anabaena *7120 cultures treated with 0.2% hexadecane. Cultures (2 L) were grown to an A _700 _= 1.0 – 1.2 prior to hexadecane addition. Fractions of 250 mL were collected at 0, 2, 12, 24, 36, 48 and 60 h after treatment. Cells were harvested by centrifugation and resuspended in 5 mL of protein buffer: 20 mM MOPS (pH 7.4), 10% glycerol, 1 mM DTT, 20 mM KCl, 1 mM PMSF, 1 mM apoprotin and 1 mM pepstatin. Samples were lysed by three passages through a French pressure cell. Soluble and membrane fractions were separated by centrifugation at 100,000 × g for 1.5 h. Pelleted membranes were resuspended in protein buffer supplemented with 0.2% deoxycholate by gentle shaking overnight at 4°C. The CYP110 protein was expressed from a pUC8 expression vector and purified as described elsewhere [16]. Antiserum to this protein was raised in a New Zealand white rabbit using subcutaneous injections of 100 μg purified CYP110 protein in Freund's complete adjuvant. Two identical booster injections were made at 3 and 6 weeks after the first. Immune serum was collect at 6 and 12 weeks after the initial injection. Protein samples (50 μg) were separated on 12% SDS-PAGE [38] and blotted using standard methods [39]. Antiserum raised against P450 protein was used at a 1:100 dilution and detected with a peroxidase-linked anti-rabbit secondary antibody (Amersham) and the ECL Western Blot system (Amersham) using X-ray film.

### Oxidized, reduced and carbon monoxide reduced spectra

The purified soluble fraction of CYP110 was diluted 1:10 in buffer A (20 mM MOPS pH 7.4, 20% Glycerol, 2 mM DTT, 20 mM KCl, and 2 mM PMSF). The oxidized spectra was measured with a Hewlett Packard Diode Array Spectrophotometer. Five milligrams of sodium dithionite was added, and the reduced spectra measured. CO was then bubbled into the reduced sample at a rate of 1 bubble per second for 30 seconds. The oxidized spectra was subtracted from the CO reduced spectra to yield the peak at 450 nm. The concentration of cytochrome P450 was determined by using the extinction coefficient, 91 mM^-1^cm^-1^, as determined for rat liver P450 [40].

### Substrate binding studies

Spectrophotometric titration of CYP110 with fatty acids, fatty amines, fatty amides, fatty alcohols, and n-alkanes with varying chain lengths (C_5_-C_20_) was preformed in buffer A with and without 0.2 % deoxycholate. Purified soluble CYP110 was diluted to a concentration of 3 μM, and the baseline spectrum was measured. Substrates were then titrated into the P450 from a 100 mM stock solution in ethanol. The n-alkanes were solubilized via the method of Scheller *et al*. [29] by sonication of the stock solution for 10 minutes on ice to enhance solubility. The substrates were titrated to concentrations varying from 5 μM to 2 mM depending upon the compound being tested. Fatty acids, amides, alcohols, esters and n-alkanes were titrated from 50 μM to 2 mM while fatty amines were titrated from 1 μM to 700 μM.

### Immunocytochemical electron microscopy

Nitrogen-fixing *Anabaena *7120 batch cultures were grown at ambient CO_2 _levels the presence or absence of 0.2% dodecane or hexadecane (v/v) under continuous light with stirring at 30 C for 30 days. Immediately upon harvesting, cells were fixed in 4% paraformaldehyde, 0.6% glutaraldehyde, 0.033 M phosphate, 0.1 M sucrose for 2 hours at 4°C. Samples were then washed three times in 7% sucrose, 0.033 M phosphate buffer. Following an ascending ethanol dehydration series (50%, 70%, 95%), the samples were infiltrated in LR white acrylic plastic (Electron Microscopy Sciences). Cells were then embedded in gelatin capsules for 24 hours at 50°C. Solidified LR-white samples were sectioned with a diamond knife and placed on carbon coated, nitrocellulose-covered, nickel grids. Sections were blocked with 1% BSA/10% goat serum in TBST (0.1% Tween, 16.5 mM Tris (pH 7.5), 137 mM NaCl, 2.7 mM KCl). The sections were incubated (2 h at room temperature) with 35 μL of the following antibodies: A. Anti-CYP110 polyclonal sera used in western blot analyses (1:400 dilution in BSA-TBST), B. preimmune rabbit IgG (1:400 dilution in BSA-TBST), or C. Immune serum preabsorbed with 2.5 mg purified CYP110 protein [16]. Following the primary antibody treatment sections were washed at least four times in BSA-TBST and incubated for one hour (room temperature) in a drop of secondary antibody goat anti-rabbit IgG (Sigma). The sections were then viewed on a Hitachi H 7000 transmission electron microscope at 80 kV.

### Conventional electron microscopy

Following growth conditions described for immunocytochemical studies, approximately 10^7 ^*Anabaena *7120 cells were pelleted and fixed by placing in 1 mL 2.5% glutaraldehyde in 0.1 M cacodylate buffer (pH 7.4) at room temperatures for 1 hour. Cells were washed twice with cacodylate buffer (15 min/wash) and embedded with 1% agar. Agar embedded samples were cut into small cubes (~1 mm) and postfixed in 1% osmium tetroxide 0.1 M cacodylate (pH 7.4) for two hours at room temperature. Samples were rinsed with deionized water and dehydrated in an ascending ethanol series. Cells were infiltrated with 50% Spurr's media (Electron Microscopy Sciences): 50% absolute ethanol, followed by at least four more infiltrations with fresh 100% Spurr's media. To allow polymerization of the Spurr's media, samples were incubated overnight at 70 C. The samples were sectioned using a diamond knife and picked up on copper grids for viewing as above.

## Authors' contributions

ST conducted the Northern and immuno-localization experiments, analyzed and organized the data and composed initial drafts for portions of the manuscript. CRF conducted the protein purification, western and substrate-binding experiments, analyzed and organized the data and composed initial drafts for portions of the manuscript. PJL conceived the project, provided overall guidance, and wrote the final version of the manuscript. All three authors read and approved the manuscript.

## Figures and Tables

**Table 1:** Spectral Shift and Binding Constants of CYP110 with SeveralCompounds.

**Compound**	**Type**	**Ks (mM)**	**Compound**	**Type**	**Ks (mM)**
Octanoic Acid	None	-	Butyl Amine	None	-
Nonanoic Acid	None	-	Pentyl Amine	II	0.54
Decanoic Acid	I	6.3	Hexyl Amine	II	0.32
Undecanoic Acid	I	5.2	Heptyl Amine	II	0.102
Dodecanoic Acid	I	1.9	Ocytl Amine	II	0.081
Tridecanoic Acid	I	0.85	Nonyl Amine	II	0.062
Tetradecanoic Acid	I	0.091	Decyl Amine	II	0.041
Pentadecanoic Acid	I	0.21	Dodecyl Amine	II	0.012
Hexadecanoic Acid	I	0.50			
Heptadecanoic Acid	I	1.1			
Octadecanoic Acid	I	3.1	12-Aminododecanoic Acid	I	0.416
Arachidonic Acid	I	0.051		I	
9-Octadecenoic Acid	I	2.8	Putrescine	None	-
9,12-Octadecadienoic Acid	I	0.72	Cetyltrimethyl Ammonium Bromide	None	-
9,12,15-Octadecatrienoic Acid	I	0.062	n-Alkanes C6-C18	None	-
11-Dodecenoic Acid	None	-			
Sodium Dodecyl Sulfate	I	-	Saturated Fatty Alcohols C6-C18	None	-
Lauryl Amide	I	2.21			
N-methyl Lauryl Amide	I	2.56	11-Hydroxydodecanoic Acid	None	-
N,N-Dimethyl Lauryl Amide	I	2.79	12-Hydroxydocecanoic Acid	None	-
Methyl Dodecanoate	None	-	N-LaurylSacosine	I	2.0

**Table 2:** Detection of anti-CYP110 polyclonal gold conjugates.

**Culture conditions**	**CYP110-gold conjugates**	
	(Avg. no. of gold particles)	
	Vegetative cell	Heterocyst cell
*Anabaena *7120 untreated	8	5
*Anabaena *7120 dodecane treatment	22	5
*Anabaena *7120 hexadecane treatment	50	5
